# An atlas of cGAS-STING signaling in pathophysiological angiogenesis and retinal vascular homeostasis across species

**DOI:** 10.1016/j.omtn.2026.102847

**Published:** 2026-01-24

**Authors:** Xuemin He, Rui Zeng, Siying Wen, Zheyao Wen, Hejun Li, Heying Ai, Rong Gao, Liwen Fan, Li Zhou, Guojun Shi, Yanming Chen, Shasha Li

**Affiliations:** 1 Department of Endocrinology and Metabolic Diseases, The Third Affiliated Hospital of Sun Yat-sen University, Guangzhou, Guangdong 510630, China; 2 Guangdong Provincial Key Laboratory of Diabetology & Guangzhou Municipal Key Laboratory of Mechanistic and Translational Obesity Research, The Third Affiliated Hospital of Sun Yat-sen University, Guangzhou, Guangdong 510630, China; 3 Department of Endocrinology and Metabolic Diseases, The Eighth Affiliated Hospital of Sun Yat-sen University, Shenzhen, Guangdong 518033, China

**Keywords:** MT: Bioinformatics, single-cell RNA-sequencing, cGAS-STING signaling, angiogenesis, VEGFA-VEGFR2 signaling, retinal vascular development, proliferative diabetic retinopathy

## Abstract

Abnormal angiogenesis is the leading cause of vision loss globally, but current anti-angiogenic treatments are unsatisfactory and incompetent. Thus, novel therapies targeting angiogenesis are urgently needed. Previously, we revealed a positive association between cGAS-STING signaling and angiogenic factors in the mouse model with ischemic retinopathy. However, whether cGAS-STING signaling regulates retinal angiogenesis remained largely unknown. Here, we analyzed single-cell RNA sequencing databases from the epiretinal fibrovascular membranes, developing mouse retinas, and normal adult retinas from *Homo sapiens*, *Sus scrofa*, and *Macaca*. Notably, we observed spatially and temporally identical expression patterns of cGAS-STING signaling and angiogenesis. In particular, cGAS-STING signaling showed the strongest correlation with angiogenesis in retinal endothelia from mice at postnatal days 3 and 6. Endothelia-specific knockout of *Sting1* in mice retarded retinal vascular growth, which was due to attenuation of VEGFA-VEGFR2 signaling as suggested by bulk RNA sequencing. In human retinal vascular endothelial cells, deletion of *STING1* prohibited VEGFR2 activation, down-regulated the levels of endothelial markers, and compromised endothelial proliferation and migration, which were counteracted by overexpression of *STING1*. This study demonstrated an evolutionally conserved interaction between cGAS-STING signaling and VEGFA-VEGFR2 signaling in pathophysiological angiogenesis and vascular homeostasis, thus providing a novel therapeutic target for treating retinal vascular diseases.

## Graphical abstract

**Figure:**
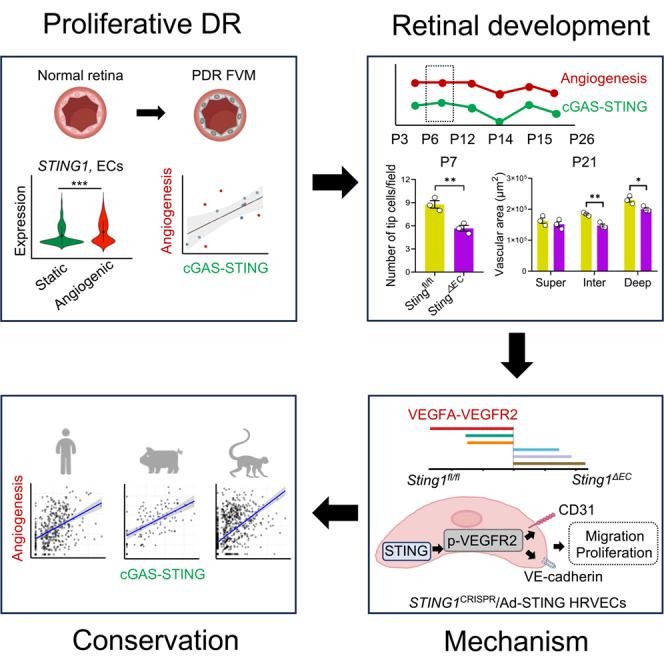

## Teaser

Endothelial cGAS-STING signaling displays a conserved interaction with the VEGFA-VEGFR2 cascade in pathophysiological angiogenesis and retinal vascular homeostasis across species. Activation of STING induced the transcription of KDR and VEGFA to promote endothelial migration and proliferation, providing a novel regulatory mechanism and therapeutic target for ocular angiogenic diseases.

## Introduction

Angiogenesis plays a central role in retinal development, which is strictly and precisely orchestrated by vascular growth factors and related signaling pathways, among which vascular endothelial growth factor A (VEGFA) signaling is the key player.^1^ VEGFA signaling controls the tip-stalk machinery in vascular network formation, as VEGF receptor 2-positive (VEGFR2^+^) tip cells respond to the stimulation of VEGFA and guide the direction of outgrowth, and VEGFR1^+^ stalk cells form the vessel lumen to extend vascular sprouting and to sustain blood perfusion.^2^ In addition to its irreplaceable functions, VEGFA-VEGFR2 signaling must coordinate with other angiogenic pathways to regulate precise vessel formation,^3^ which is largely unclear under pathophysiological conditions.

Proliferative diabetic retinopathy (PDR) is characterized by aberrant angiogenesis and outgrowth of the epiretinal fibrovascular membrane (FVM), which has become a leading cause of vision loss in diabetic patients.^4^ Single-cell RNA sequencing (scRNA-seq) revealed that the cell clusters of the FVM are identical to retinal cells,^5^ suggesting that the FVM likely originates from the retina. However, the gene expression patterns in the FVM cells differ from those in normal retinal cells and diabetic retinal cells.^6^ Yet what mechanisms trigger this alteration remain to be studied.

Cyclic GMP-AMP synthase (cGAS) senses endogenous or exogenous DNA from different sources, which activates the stimulator of interferon genes (STING) and triggers innate immune responses.^7^ Recently, we and other groups demonstrated activation of cGAS-STING signaling in diabetic retinal vessels, promoting inflammation and senescence in endothelial cells (ECs).^6^^,^^8^ In addition, cGAS-STING signaling may participate in retinal angiogenesis, as knockout of *Sting1* in myeloid cells or systemic inhibition of STING protein ameliorated retinal angiogenesis and vascular leakage in the ischemic retinopathy^9^^,^^10^ and age-related macular degeneration^11^ models. Notably, we also noticed an enrichment of *STING1* mRNA in angiogenic ECs from the FVM, and endothelial *Sting* expression was positively correlated with the transcriptional levels of angiogenic factors in ischemic retinas.^12^ However, how cGAS-STING signaling regulates the angiogenic process remains largely unknown.

In this study, we aimed to illustrate the functions of cGAS-STING signaling in retinal angiogenesis under physiological and pathological conditions. By analyzing the scRNA-seq databases of the FVM, developing mouse retinas, and normal retinas from *Homo sapiens*, *Sus scrofa*, and *Macaca*, we validated a conserved interaction of cGAS-STING signaling with angiogenesis in the retinas across species. Moreover, EC-specific *Sting1* knockout (*Sting1*^ΔEC^) mice displayed retarded retinal vascular sprouting and formation, which was due to attenuation of VEGFA-VEGFR2 signaling as suggested by bulk RNA-seq. In human retinal vascular ECs, deletion of *STING1* prohibited VEGFR2 activation, down-regulated the levels of endothelial markers, and compromised endothelial proliferation and migration, which were counteracted by overexpression of *STING1*. In summary, this study demonstrated that cGAS-STING signaling regulated pathophysiological angiogenesis and vascular homeostasis via the modulation of VEGFA-VEGFR2 signaling, which may represent a novel therapeutic target for retinal vascular diseases.

## Results

### *STING1* is enriched in angiogenic ECs from the PDR FVM

**Figure 1:**
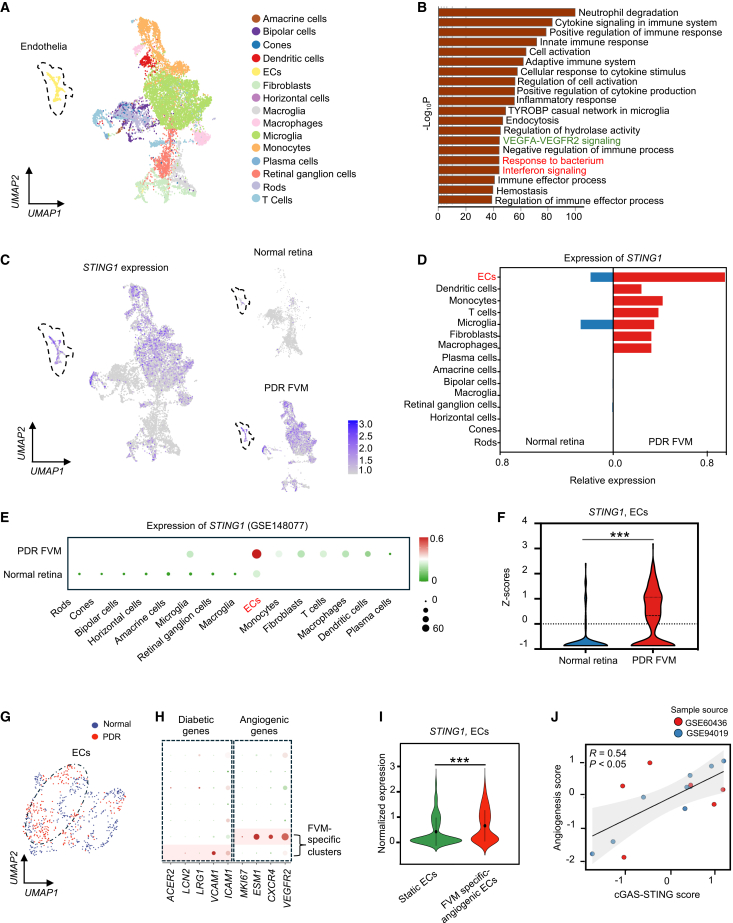
Enrichment of *STING1* in angiogenic ECs from the PDR FVM (A) UMAP showing the integrated cell populations from normal human retinas and the PDR FVM. The dashed box indicates ECs. (B) Bar plots of the top 20 significantly changed biological functions in the PDR FVM compared to normal retinas. (C) UMAP showing *STING1* expression in the integrated, normal, and PDR populations. (D) Bidirectional bar chart illustrating the ranking of *STING1* expression in the cells from the PDR FVM and normal retinas. (E) Expression of *STING1* across the cell types in normal retina and the PDR FVM (GSE148077). (F) Comparison of the relative levels of *STING1* in the ECs from the PDR FVM and normal retinas. (G) Comparison of *STING1* expression levels in angiogenic and non-angiogenic ECs from the PDR FVM. UMAP visualization of ECs of the PDR FVM and healthy retinas. (H) Dot plot showing diabetic and angiogenic gene expression in EC subclusters. (I) Violin plot showing *STING1* expression in FVM-specific angiogenic ECs compared with static ECs. (J) Spearman correlation analysis (R score) and correlation test (*p* value) between gene signatures of angiogenesis and cGAS-STING signaling in the ECs from the PDR FVM. ∗∗∗*p* < 0.001.

The FVM may originate from the retina,^5^ but the mechanism regulating its occurrence and outgrowth was largely unknown. Our previous study demonstrated enrichment of *STING1* in the ECs from the FVM,^12^ whether cGAS-STING signaling played a role in promoting the FVM remained to be studied. First, we integrated two public scRNA-seq datasets of normal healthy retinas and the FVM from PDR patients.^5^^,^^13^ In total, we obtained 11,143 single cells, including 3,248 cells from normal retinas and 7,895 cells from the FVM (Figures S1A and S1B). In the integrated dataset, we identified 27 transcriptionally distinct clusters (Figure S1C) and 15 distinct cell types, including rod photoreceptor cells, cone photoreceptor cells, amacrine cells, astrocytes, bipolar cells, horizontal cells, microglia, Müller glia, retinal ganglion cells, and ECs (Figure 1A). When comparing the gross gene expression in the PDR FVM to that in normal retinas, inflammatory pathways and VEGFA-VEGFR2 signaling were ranked as the top 20 signaling pathways. Surprisingly, STING-related signaling “response to bacterium” and “interferon signaling” were also identified (Figure 1B), suggesting an important role of cGAS-STING signaling in the pathology of the FVM.

First, we analyzed the expression pattern of the cGAS-STING pathway in the PDR FVM. Notably, ECs showed a marked increase in *STING1* expression. Analysis of two independent cohorts (GSE148077 and GSE137846) confirmed that *STING1* was not only up-regulated in ECs derived from in PDR FVM but also expressed at relatively higher level than other cell types within the FVM (Figures 1C–1E and S1F). Moreover, when comparing the expression specifically in the ECs, the FVM displayed much higher expression of *STING1* relative to normal retinas (Figure 1F).

Given the crucial role of angiogenesis in the FVM outgrowth, we wondered if endothelial cGAS-STING signaling played a role in the angiogenic process. Using a multi-marker approach (*KDR*, *CXCR4*, *DLL4*, *MKI67*, etc.) to define angiogenic ECs, we identified a pro-angiogenic subcluster within the FVM that exhibited significantly elevated *STING1* expression compared to static ECs (Figures 1G–1I and S1G–S1I). Moreover, bulk RNA-seq results of two datasets showed a positive correlation of angiogenesis and cGAS-STING signaling (R = 0.54, *p* < 0.05) (Figure 1J). Collectively, these data suggest the involvement of cGAS-STING signaling in the pathological angiogenesis of the FVM.

### Endothelial cGAS-STING signaling is positively correlated with physiological angiogenesis throughout retinal development

**Figure 2:**
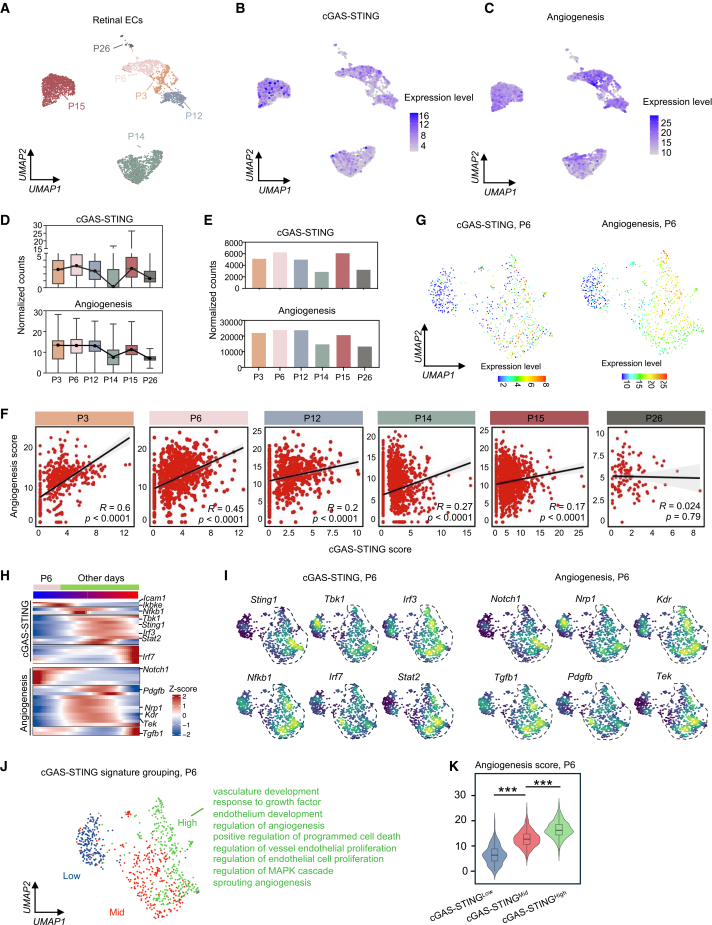
Positive association of *Sting1* expression with physiological angiogenesis in the ECs throughout retinal development (A) UMAP of retinal ECs merged from P3 to P26 mice. (B and C) UMAP showing the gene signatures of cGAS-STING signaling and angiogenesis in the mouse retinal ECs from different developmental stages. (D and E) Boxplot illustration and total scores of the gene signatures of cGAS-STING signaling and angiogenesis in the mouse retinal ECs across developmental stages. The cell count at each developmental stage was normalized to the maximum cell count. (F) Spearman correlation analysis (R score) and correlation test (*p* value) between gene signatures of angiogenesis and cGAS-STING signaling in the retinal ECs across developmental stages. (G) UMAP of the gene signatures of cGAS-STING signaling and angiogenesis in the retinal ECs from P6 mice. (H and I) Pseudotime heatmap showing the individual gene expression, and UMAP of cGAS-STING signaling and angiogenesis in the retinal ECs from P6 mice and other days. (J) UMAP plot of ECs grouped by cGAS-STING signature levels at the P6 retina. (K) Violin plots showing angiogenesis scores in the retinal ECs with increased cGAS-STING activity at P6. ∗∗∗*p* < 0.001.

To evaluate whether cGAS-STING signaling played a role in physiological angiogenesis, we investigated scRNA-seq datasets of mouse retinas at various postnatal days (P) including P3, P6, P12, P14, P15, and P26. A total of 5,562 retinal ECs were integrated for subsequent analysis (Figure 2A). In the retinal ECs throughout the developmental stages, the gene signatures of cGAS-STING signaling and angiogenesis displayed a similar activation trend (Figures 2B, 2C, and S2A–S2C). In particular, the cGAS-STING score peaked at P6 (Figures 2D and 2E), implying that this signaling is highly active during the early stages of retinal vascular development. Interestingly, the angiogenesis score exhibited a similar trend from P3 to P26 (Figures 2D and 2E). Correlation analysis further demonstrated a strong positive association between the gene signatures of cGAS-STING signaling and angiogenesis from P3 to P15, particularly at P3 and P6 (R = 0.6 and 0.45, respectively) (Figures 2F, S2D, and S2E). Moreover, to directly address angiogenic EC heterogeneity in developing retinas, we re-clustered the redefined angiogenic ECs and identified five distinct subclusters. These subclusters showed differential marker expression patterns (e.g., *Dll4/Cxcr4* and *Vegfr2/Flt1/Aplnr*) and distinct pathway enrichments (e.g., AP-1 pathway, VEGFA-VEGFR2 pathway, chromatin remodeling, extracellular matrix organization, cell cycle, and cGMP-PKG signaling pathway) (Figures S2F–S2H). Notably, the correlation disappeared at P26, coinciding with the completion of retinal vascular angiogenesis at around P26 in murine models.^14^

Given that the cGAS-STING signature peaked at P6, we further investigated the expression of cGAS-STING signaling and angiogenesis in the ECs at this time point. The endothelial cluster was pulled out (Figures S3A–S3C). As expected, the concurrent expression pattern of these two signaling pathways was identified (Figure 2G). Further analyses of the dynamic and landscape expression of individual genes also confirmed the same conclusion (Figures 2H and 2I). Compared with other time points, P6 gradually gained expression of the genes from cGAS-STING signaling and angiogenesis (Figure 2H). Overall, these results indicate that the expression of cGAS-STING signaling was positively correlated with that of angiogenesis in retinal ECs across development stages. To directly assess the functional relevance, we stratified P6 ECs into low, mid, and high groups based on the cGAS-STING pathway activity (Figures 2J and S3D). Gene Ontology analysis revealed that the cGAS-STING^High^ subset was selectively enriched for angiogenesis-related processes, including “vascular development,” “regulation of endothelial cell proliferation,” and “sprouting angiogenesis” (Figure S2H). Correspondingly, angiogenesis scores increased progressively from the low to the high group, confirming a positive association between cGAS-STING activation and pro-angiogenic transcriptional programs (Figure 2K).

### Specific knockout of endothelial *Sting1* retards the retinal vascular development

**Figure 3:**
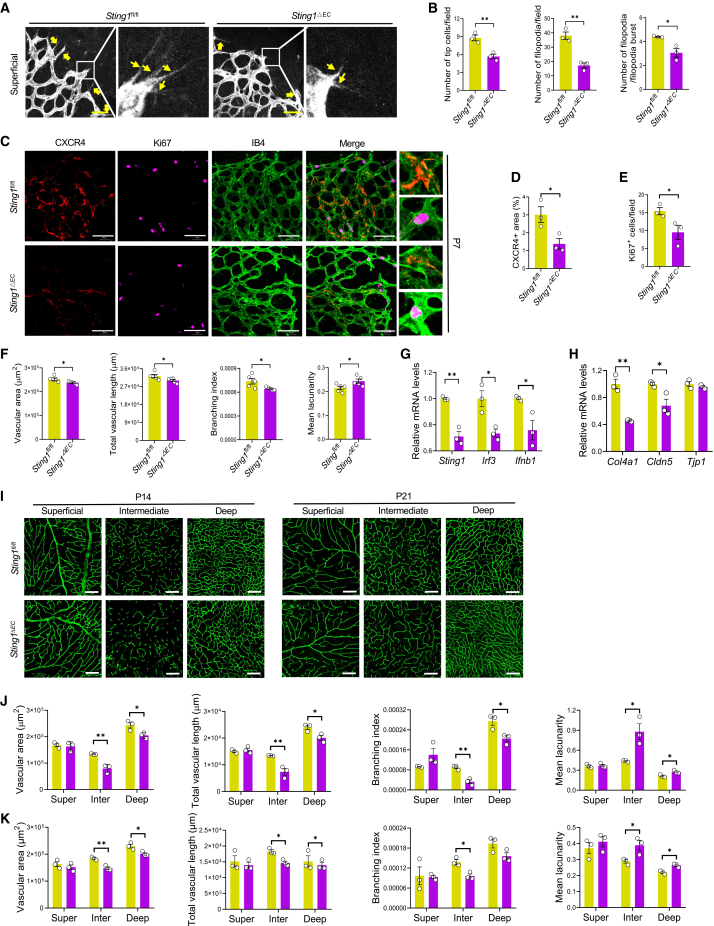
Delayed retinal vascular growth by *Sting1* deficiency in the retinal ECs (A and B) Representative images of *Sting1*^ΔEC^ retinal flatmounts stained by IB4 at P7, and quantification of the numbers of tip cells, number of filopodia, and the ratio of filopodia number to filopodia burst (*N* = 3). Scale bars, 50 μm. Yellow arrows denote filopodia burst. (C–F) Representative images of the superficial layer in *Sting1*^ΔEC^ retinal flatmounts stained by IB4, CXCR4, and Ki67 at P7, and quantification of CXCR4^+^ area, Ki67^+^ cells/field, vascular areas, total vascular lengths, branching index, and mean lacunarity (*N* = 3–5). Scale bars, 100 μm. (G and H) Quantification of the mRNA levels of cGAS-STING signaling components, including *Sting1*, *Irf3*, and *Ifnb1*, and junctional components *Col4a1*, *Tjp1* and *Cldn5* in *Sting1*^ΔEC^ retinal flatmounts at P21 (*N* = 3). (I–K) Representative images of the superficial, intermediate, and deep layers in *Sting1*^ΔEC^ retinal flatmounts stained by IB4 at P14 and P21, and quantification of vascular areas, total vascular lengths, branching index, and mean lacunarity (*N* = 3). Scale bars, 100 μm. *Sting1*^fl/fl^ retinal flatmounts were used as the control. ∗*p* < 0.05, ∗∗*p* < 0.01.

To experimentally validate the impact of cGAS-STING signaling on physiological angiogenesis across development stages, we generated a mouse strain with EC-specific knockout of *Sting1* (*Sting1*^ΔEC^). Flow cytometry demonstrated significant reduction of STING protein levels in CD31^+^ cells, but not CD45^+^ cells, from *Sting1*^ΔEC^ retinas compared to *Sting1*^flox/flox^ (*Sting1*^fl/fl^) controls (Figures S5B and S5C). The formation of retinal vasculature does not begin until birth in murines,^2^ and the superficial plexus is the first to develop, which starts after birth and completes at around P8.^14^ Tip cells are induced by the balance between angiogenic and angiostatic factors at the forefront of sprouting vessels, which sense the adjacent environment by protruding specialized filopodia under the guidance of astrocyte.^15^ Thus, we measured the number of tip cells and filopodia in the retinas of P7 pups by staining isolectin *Griffonia simplicifolia* lectin I isolectin B4 (IB4). Compared to those of *Sting1*^fl/fl^ littermates, the numbers of tip cells, filopodia/field, and filopodia/filopodia bursts were significantly reduced in the retinas of *Sting1*^ΔEC^ mice (Figures 3A and 3B). Consistently, the vascular areas, total vascular length, and branching index were also consistently lower. In contrast, the mean lacunarity, which characterizes vessel non-uniformity, was increased in the superficial plexus of these *Sting1*^ΔEC^ retinas (Figures 3C and 3D). Moreover, the expression of CXCR4, a critical regulator of vascular angiogenesis,^16^ was less in the vascular forefronts of *Sting1*^ΔEC^ retinas, which was accompanied with less Ki67-positive cells (Figures 3C and 3D).

The formation of the deeper plexus starts at P8 and that of the intermediate plexus at P10.^14^ When comparing the deep plexus, only the vascular areas and total vascular length at P14 were less in the retinas of *Sting1*^ΔEC^ mice than those in *Sting1*^fl/fl^ mice; by P21, the development of the deep plexus in *Sting1*^fl/fl^ mice caught up with that of control mice (Figures 3E–3G). Regarding the intermediate plexus, the vascular areas, total vascular length, and branching index were consistently lower, whereas the mean lacunarity was increased in *Sting1*^ΔEC^ retinas at P14 and P21 (Figures 3E–3G). Interestingly, there is no difference in the superficial plexus by P14. Furthermore, we examined the molecular changes of the P21 retinas and found significantly lower levels of tight junction components *Col4a1*, *Tjp1*, and *Cldn5* (Figure 3I) in P21 *Sting1*^ΔEC^ mice, which was accompanied by less expression of *Sting1*, transcriptional factor *Irf3*, and downstream target gene *Ifnb1* (Figure 3H). The above results suggested retarded retinal angiogenesis by *Sting1* deficiency during retinal development.

### Deficiency of *Sting1* compromises the activation of VEGFA-VEGFR2 signaling in the retinal vasculature

**Figure 4:**
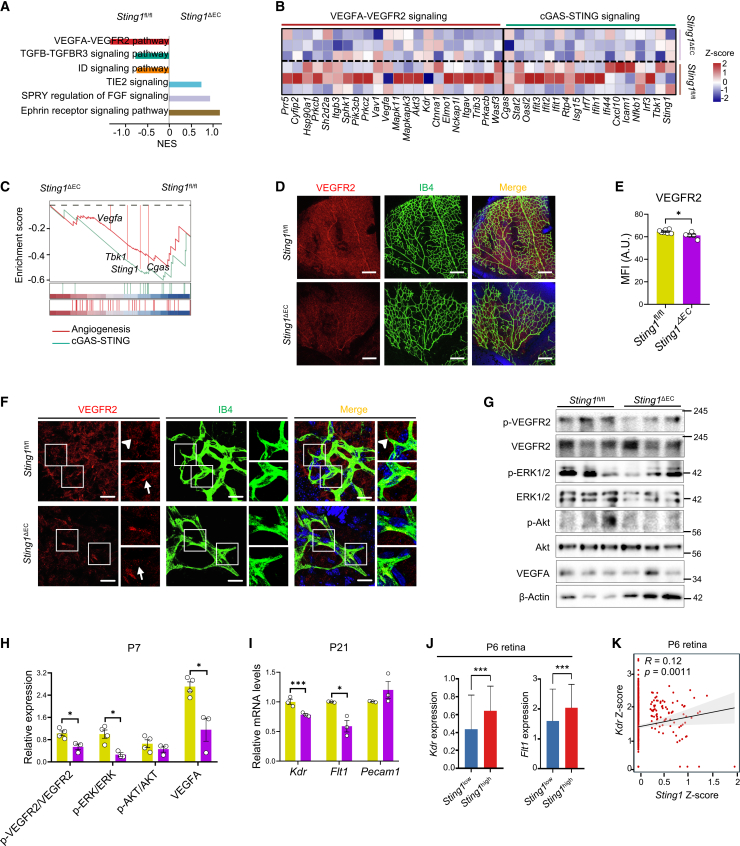
Attenuation of VEGFA-VEGFR2 signaling in the retina of *Sting1*^ΔEC^ mice (A) Bar plots depicting the most significantly altered angiogenic pathways in *Sting1*^ΔEC^ retinas at P7. (B and C) Heatmap of selected genes, and GSEA of cGAS-STING and VEGFA-VEGFR2 signaling in *Sting1*^ΔEC^ retinas (*N* = 3). (D–F) Immunostaining and quantification of VEGFR2 in *Sting1*^ΔEC^ retinal flatmounts at P7 (*N* = 5–7). Scale bars, 100 or 10 μm. (G and H) Western blot analysis and densitometry quantification of p-VEGFR2, VEGFR2, p-EKR1/2, ERK1/2, p-AKT, AKT, and VEGFA in retinal lysates from *Sting1*^ΔEC^ retinas at P7 (*N* = 3–4). (I) Quantification of the mRNA levels of *Kdr*, *Flt1*, and *Pecam1* in *Sting1*^ΔEC^ retinas at P21 (*N* = 3). (J) Comparison of the mRNA levels of *Kdr* and *Flt1* in *Sting1*^high^ retinal cells relative to *Sting1*^low^ retinal cells from P6 mice. (K) Spearman correlation analysis (R score) and correlation test (*p* value) of *Sting1* and *Kdr* in the retinal cells from P6 mouse. *Sting1*^fl/fl^ retinas were used as the control. ∗*p* < 0.05, ∗∗∗*p* < 0.001.

To understand the molecular mechanisms underlying the impact of *Sting1* deficiency in retinal vessels, we isolated retinas from P7 mice for bulk RNA-seq analysis. Results showed that compared to *Sting1*^fl/fl^ controls, the counts of angiogenesis and expression of individual genes from hallmark angiogenesis were decreased in *Sting1*^ΔEC^ retinas (Figure S4). Gene set enrichment analysis (GSEA) further demonstrated significant enrichment of angiogenic pathways in the retinas from *Sting1*^ΔEC^ mice. In particular, VEGFA-VEGFR2 signaling, TGFB-TGFBR3 signaling, and Inhibitor of DNA-binding (ID) signaling were down-regulated, whereas TIE2 signaling, SPRY-FGF signaling, and ephrin receptor signaling were up-regulated (Figure 4A). Notably, VEGFA-VEGFR2 signaling ranked as the most significantly changed pathway. We further examined individual gene expression from VEGFA-VEGFR2 signaling. In alignment with the changes in cGAS-STING signaling, reduced levels of genes of VEGFA-VEGFR2 signaling were found in *Sting1*^ΔEC^ mice (Figure 4B). We further compared and found similar GSEA trends of VEGFA-VEGFR2 signaling and cGAS-STING signaling in *Sting1*^ΔEC^ retinas (Figure 4C).

To confirm the changes in VEGFA-VEGFR2 signaling, we performed immunostaining of VEGFR2 in the retinal flatmounts from P7 mice. At the angiogenic fronts, VEGFR2^+^ tip cells sense high concentrations of VEGFA, which, in turn, down-regulates the expression of VEGFR2 while up-regulating VEGFR1 in stalk cells.^2^ The overall levels of VEGFR2 were lower in *Sting1*^ΔEC^ retinas compared to controls (Figures 4D and 4E). Particularly, VEGFR2 was enriched in mature vessels and tip cells; however, knockout of *Sting1* disrupted the distribution patterns of VEGFR2 in retinal vessels (Figure 4F). Western blot analysis further confirmed attenuation of VEGFA-VEGFR2 signaling as indicated by reduced protein levels of p-VEGFR2/VEGFR2, p-ERK/ERK, and VEGFA (Figures 4G and 4H). Surprisingly, even by P21 when the retinal vasculature of *Sting1*^ΔEC^ mice seemed to catch up with that of littermates, the transcriptional levels of *Kdr,* a gene that encodes VEGFR2 and serves as a specific marker for tip cells and neural retina,^17^ and *Flt1*, encoding VEGFR1, which is highly expressed in mature vessels,^18^ were still significantly less in *Sting1*^ΔEC^ retinas (Figure 4I). Notably, *Pecam1* levels were not altered, suggesting that endothelial *Sting1* deficiency did not affect retinal endothelial number.

Analysis of the scRNA-seq data from P6 mouse retinas further showed that *Sting1*^high^ retinal cells possessed higher levels of *Kdr* and *Flt1* (Figure 4J). Consistently, *STING1*^high^ cells from the PDR FVM displayed increased expression of *KDR*, *FLT1*, and *VEGFA* (Figure S1D). In addition, positive associations of the transcriptional levels of STING and VEGFR2 were detected in the ECs from P6 mouse retinas and the PDR FVM (R = 0.12 and 0.83, respectively) (Figures 4K and S1E). Taken together, these data identified a novel regulatory function of cGAS-STING signaling on VEGFA-VEGFR2 signaling under physiological and pathological angiogenesis.

### STING modulates the proliferation and migration of retinal vascular ECs *in vitro*

**Figure 5:**
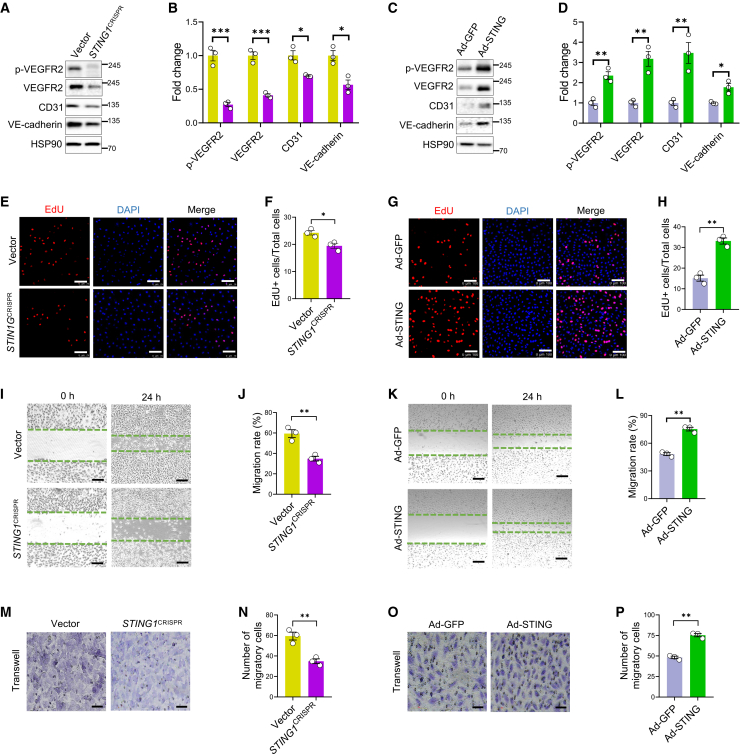
Regulation of the angiogenic activities of retinal vascular ECs by STING (A–D) Western blot analysis and densitometry quantification of VEGFA-VEGFR2 signaling components p-VEGFR2 and VEGFR2, endothelial markers CD31, and tight junctional component VE-cadherin in *STING1*^CRISPR^ HRVECs and HRVECs after infection with Ad-STING for 48 h (*N* = 3). (E–H) Representative images and quantification of EdU^+^ cells in *STING1*^CRISPR^ HRVECs and HRVECs after infection with Ad-STING for 48 h (*N* = 3). Scale bars, 100 μm. (I–P) Representative images and quantification of wound closure (scale bars, 200 μm) and migratory cells stained by violet crystal (scale bars, 500 μm) in *STING1*^CRISPR^ HRVECs and HRVECs after infection with Ad-STING for 48 h (*N* = 3). Vector HRVECs or Ad-GFP were used as the controls. ∗*p* < 0.05, ∗∗*p* < 0.01, ∗∗∗*p* < 0.001.

To confirm the regulatory effect of cGAS-STING on VEGFA-VEGFR2 signaling, we utilized a human retinal vascular EC (HRVEC) cell line, whose endothelial lineage was confirmed by immunostaining of CD31 (Figure S6E). We further generated a stable *STING1-*deficient strain based on the CRISPR-Cas9 system (*STING1*^CRISPR^)^6^ and tested the knockout efficiency of *STING1* by Western blot analysis (Figures S6A and S6B). Consistently, the levels of p-TBK1, essential for STING trafficking from the endoplasmic reticulum to the Golgi,^19^ and downstream transcriptional factors p-p65 and p-IRF3, were dramatically decreased (Figures S6A and S6B). We then tested the status of VEGFA-VEGFR2 signaling. As expected, a significant reduction of p-VEGFR2 was detected (Figures 5A and 5B), consistent with the attenuated VEGFA-VEGFR2 signaling in *Sting1*^ΔEC^ retinas. Moreover, compromised endothelial integrity was also identified as indicated by decreases in CD31 and VE-cadherin (Figures 5A and 5B). On the other hand, treatment of an adenovirus overexpressing human *STING1* (Ad-STING) augmented the ratios of p-TBK1/TBK1, p-p65/p65, and p-IRF3/IRF3 in HRVECs (Figures S6C and S6D), followed by up-regulation of p-VEGFR2, VEGFR2, CD31, and VE-cadherin (Figures 5C and 5D). These results demonstrated that STING indeed regulates the activation of VEGFA-VEGFR2 signaling.

We further examined the impact of cGAS-STING signaling on retinal vascular ECs *in vitro*. Knockout of *STING1* compromised the proliferative ability of HRVECs, as indicated by less incorporation of EdU (Figures 5E and 5F). Conversely, when cells were treated with Ad-STING, the proliferative capacity was enhanced (Figures 5G and 5H). Additionally, wound closure and transwell assays showed reduced migration in *STING1*^CRISPR^ HRVECs, which was increased in HRVECs treated with Ad-STING (Figures 5I–5P). The above results confirmed that cGAS-STING signaling potentiated the activation of VEGFA-VEGFR2 signaling to induce the proliferation and migration of retinal ECs.

### VEGFA-VEGFR2 signaling is the downstream cascade of STING in angiogenic regulation

**Figure 6:**
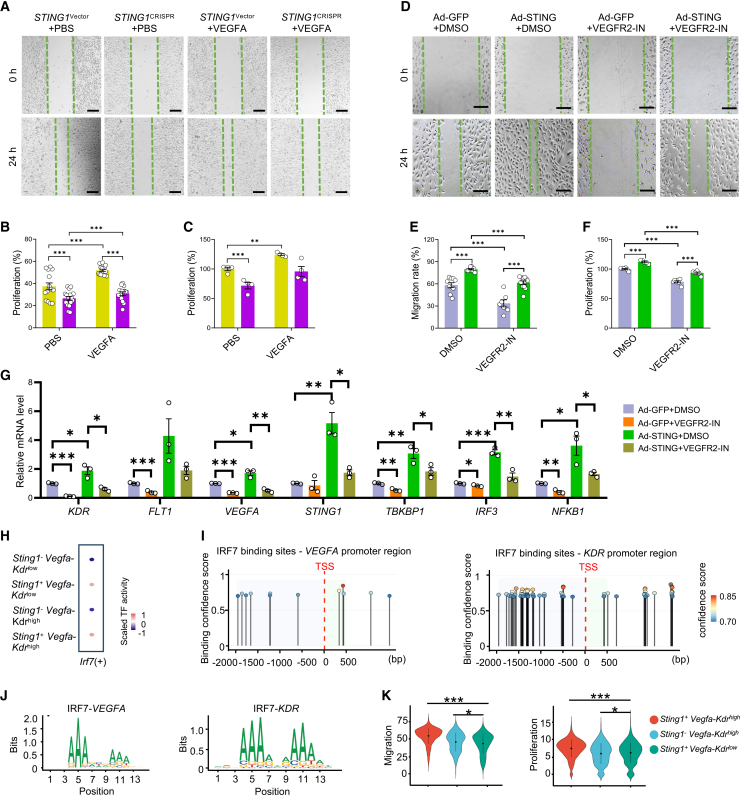
VEGFA-VEGFR2 signaling as the downstream cascade of STING in agiogenic regulation (A–C) Representative images and quantification of wound closure (scale bars, 200 μm), and proliferation rate in HRVECs after infection with Ad-STING for 48 h, followed by treatment of VEGFR2-IN (40 μM) for 24 h (*N* = 7–12). (D–F) Representative images and quantification of wound closure (scale bars, 100 μm), and proliferation rate in *STING1*^CRISPR^ HRVECs treated with VEGFA (50 ng/mL) for 24 h (*N* = 7–12). (G) Quantification of the mRNA levels of *KDR*, *FLT1*, *VEGFA*, *STING1*, *TBKBP1*, *IRF3*, and *NFKB1* in HRVECs after infection with Ad-STING for 48 h, followed by treatment of actinomycin-D (2 μM) for 24 h (*N* = 3). (H) Dot plot illustrating differential IRF7 activity among developing retinal EC subpopulations defined by *Sting1* and *Vegfa-Kdr* expression levels. (I) Predicted IRF7-binding sites within the *VEGFA* and *KDR* promoter regions. (J) Sequence logos depicting IRF7-binding motifs for the *VEGFA* and *KDR*. (K) Enrichment scores for cell migration and proliferation of developing retinal ECs under three conditions: *Sting1*^*+*^*Vegfa*^*-*^*Kdr*^high^, *Sting1*^*-*^*Vegfa*^*-*^*Kdr*^high^, and *Sting1*^*+*^*Vegfa*^*-*^*Kdr*^low^. Vector HRVECs or Ad-GFP were used as the controls. ∗, *p* < 0.05, ∗∗, *p* < 0.01, ∗∗∗, *p* < 0.001.

To further confirm whether STING regulates angiogenic activities in ECs through the mediation of VEGFA-VEGFR2 signaling, we stimulated *STING1*^CRISPR^ HRVECs with VEGFA, but failed to find a notable migratory and proliferative response to VEGFA (Figures 6A–6C). This result confirmed that *STING1* deficiency compromised the angiogenic activities of retinal ECs. Additionally, we utilized a VEGFR2 inhibitor (VEGFR2-IN) in HRVECs with STING overexpression. The wound closure and proliferation assays both demonstrated that VEGFR2-IN efficiently blocked the potentiated angiogenic effect of STING overexpression (Figures 6D–6F).

To further understand how STING regulates VEGFA-VEGFR2 signaling, we treated cells with actinomycin-D to abolish transcription activity. As shown in Figure 6G, STING overexpression significantly increased the mRNA levels of *KDR*, *FLT1*, and *VEGFA*, accompanied by up-regulation of *STING1*, *TBKBP1*, *IRF3*, and *NFKB1*, whereas actinomycin-D treatment blunted the inductive effect of STING overexpression. Furthermore, cycloheximide was applied to block protein translation of VEGFR2. Although STING overexpression increased the protein levels of VEGFR2, it did not affect the degradation rate of VEGFR2 (Figure S6F).

Additionally, our scRNA-seq analysis using PySCENIC revealed that IRF7, a well-established transcription factor within the cGAS-STING pathway, was highly enriched in the cell populations co-expressing *Kdr* and *Sting1* (Figure 6H). Moreover, Motif enrichment and genome-wide binding site analyses identified conserved IRF7-binding motifs within the promoter regions of *KDR* and *VEGFA* (Figures 6I and 6J). We also performed additional single-cell transcriptomic analyses focusing on ECs stratified by both STING activity and VEGFA-VEGFR2 signaling scores. Notably, cells exhibiting high expression of both *Sting1* and *Kdr* showed markedly elevated migration and proliferation scores compared with those exhibiting high expression of only one of these factors (Figure 6K). These results together suggested that STING regulates VEGFA-VEGFR2 signaling through transcriptional level. These results provide transcriptomic evidence supporting that STING promotes endothelial proliferation and migration primarily through the VEGFA-VEGFR2 signaling axis, consistent with the mechanistic rationale underlying the reviewer’s suggested inhibition experiments.

### CGAS-STING signaling regulates vascular homeostasis in the adult retinas across species

**Figure 7:**
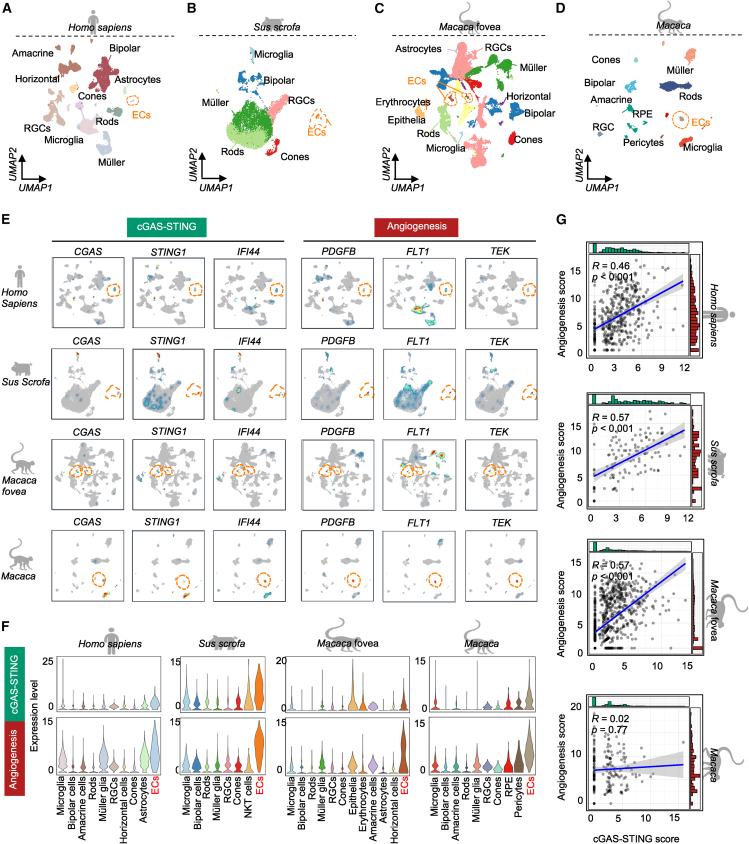
Association of cGAS-STING signaling with angiogenesis in the vascular homeostasis of adult retinas across species (A–D) UMAP of retinal cell types from *Homo sapiens*, *Sus scrofa*, *Macaca* fovea, and *Macaca*. The dashed box indicates ECs. (E) UMAP showing the expression levels of selected genes from cGAS-STING signaling and angiogenesis in the retinal cells of *Homo sapiens*, *Sus scrofa*, *Macaca* fovea, and *Macaca*. The dashed box indicates ECs. (F) Violin plots illustrating the expression signatures of cGAS-STING signaling and angiogenesis in the retinal cells across species. (G) Spearman correlation analysis (R score) and correlation test (*p* value) between the gene signatures of angiogenesis and cGAS-STING signaling in the retinal ECs.

Hitherto, we have demonstrated that cGAS-STING signaling regulated pathological and physiological angiogenesis in humans and mice; whether it also plays a role in vascular homeostasis in the adult retinas across species remains unknown. To comprehensively explore the function of cGAS-STING signaling under this condition, we investigated public scRNA-seq datasets of the adult retinas from *Homo sapiens*,^20^ *Sus scrofa*,^21^ and *Macaca* (fovea^22^ and whole retina^23^). Similar retinal cell types were identified in these species (Figures 7A–7D). Then, we explored the gene expression of cGAS-STING signaling and angiogenesis in each species. Notably, we observed that *CGAS*, *STING1*, and *IFI44* from cGAS-STING signaling, and *PDGFB*, *FLT1*, and *TEK* from angiogenesis, were highly and specifically expressed in the ECs (Figure 7E). Additionally, we evaluated the profiles of gene signatures of these two pathways in different cell types. The plots showed that the retinal ECs expressed the highest levels of these two pathways across species (Figure 7F), confirming their crucial role in retinal vascular homeostasis. We also observed a positive significant correlation between cGAS-STING and angiogenesis (R = 0.46–0.57) in the retinal ECs of *Homo sapiens*, *Sus sucrofa*, and *Macaca* fovea (Figure 7G). In summary, our study demonstrated an evolutionary function of cGAS-STING signaling in maintaining retina vascular homeostasis across species.

## Discussion

Physiological angiogenesis is fundamental for retinal development, but abnormal angiogenesis is the most severe cause of vision loss, affecting a large population from the elderly to the newborn, particularly in developed countries. However, current anti-angiogenic treatments lack satisfactory efficacy and are accompanied by many side effects due to the complex interaction of angiogenic pathways. Therefore, it is urgent to explore novel therapies to treat this globally prevalent disease.

It is well known that cGAS-STING signaling is a pathogenic factor in vasculopathy^24^ and cardiovascular diseases.^25^ Recently, we and others have observed abnormal activation of cGAS-STING signaling in DR,^6^^,^^9^^,^^12^^,^^26^ age-related macular degeneration,^27^ and ischemic retinopathy,^10^^,^^26^ promoting the secretion of inflammatory factors, endothelial senescence, and capillary degeneration. Moreover, we found a positive correlation of *Sting1* expression with angiogenic factors in the ECs of ischemic retinas,^12^ suggesting a potential connection of cGAS-STING with retinal angiogenesis. In this study, we analyzed the scRNA-seq data of the retinas of DR and PDR and found identical expression patterns of cGAS-STING signaling and pathological angiogenesis (Figure 1). Due to its dual functions in regulating inflammation and angiogenesis, cGAS-STING signaling seems a good candidate for treating ocular diseases. We and others confirm that inhibition of cGAS-STING signaling rescues vascular dysfunction and endothelial senescence in DR.^8^^,^^12^ Intravitreal injections of thermal-sensitive hydrogel or phosphatidylserine-modified nanoparticles, which are loaded with STING inhibitors, effectively antagonize ocular inflammation and prohibit retinal angiogenesis.^28^^,^^29^ The supernatant from macrophages treated by STING agonists potently impairs the migratory capacities of vascular ECs.^30^ Moreover, genetic knockout of the *Cgas* gene in myeloid cells inhibits retinal angiogenesis in an ischemic model.^10^ Deletion of *Sting1* significantly suppresses the pathological angiogenesis in the models of choroidal neovascularization and ischemic retinopathy.^26^ Taken together, cGAS-STING signaling is a promising therapeutic target for angiogenic diseases.

Regarding the role of cGAS-STING signaling in physiological angiogenesis, global *Cgas* deficiency has been reported to impair angiogenesis and vascular disruption in zebrafish.^31^ Deletion of endothelial *Sting1* disrupts vascular angiogenesis and oligodendrogenesis in embryonic mouse forebrains.^32^ This study analyzed the scRNA-seq data of developing mouse retinas and demonstrated strong positive correlations of cGAS-STING signaling and angiogenesis from P3 to P15 (Figure 2F). Additionally, by generating *Sting1*^ΔEC^ mice, we detected retarded vascular development in the *Sting1*^ΔEC^ retinas at P7 to P14 (Figure 3). Results of *STING1*^CRISPR^ HRVECs and Ad-STING further showed alterations in endothelial proliferation and migration (Figure 5). Therefore, this study identified a novel regulatory function of cGAS-STING signaling on the physiological angiogenic process, expanding our understanding of the complicated orchestration of angiogenic systems under conditions such as tissue repair and wound healing.

It has been reported that activation of cGAS-STING signaling up-regulates the levels of VEGFA in retinal pigment epithelium,.^33^ whereas STING inhibitors down-regulate VEGFA expression in murine myeloid cells.^34^ In addition, nuclear cGAS is proven to be indispensable for the angiogenic activity of VEGFA.^31^ In this study, we revealed attenuated VEGFA-VEGFR2 signaling in the *Sting1*^ΔEC^ retinas at P7 as suggested by bulk RNA-seq analysis (Figure 4A). Enriched expression of VEGFR2 was detected in the tip cells of normal retinas, whereas *Sting1* deficiency disrupted this enrichment and down-regulated the protein levels of VEGFR2 in P7 retinas (Figures 4D–4F). Moreover, we observed lower transcriptional levels of *Kdr* and *Flt1* in the *Sting1*^ΔEC^ retinas, when the vasculature seemed to catch up compared to controls (Figure 4I), suggesting a direct regulation of VEGF receptors by cGAS-STING signaling. However, it remains unclear whether this regulation depends on canonical cGAS-STING signaling, that is, through the mediation of transcriptional factors including p-IRF3 or p-p65.

Previous studies have revealed antagonizing effects of interferon (IFN) signaling in endothelial angiogenesis. For instance, IFN members, including IFN-α, IFN-β, and IFN-γ, were shown to blunt the VEGFA signaling cascade,^35^ prohibit EC proliferation and tubular formation,^36^^,^^37^ induce the secretion of CXCL9 to suppress vascular angiogenesis,^38^ and alleviate the progression of DR and other ocular diseases.^39^ Moreover, degradation of IFN receptor resulted in enhanced angiogenic activities induced by VEGFA in HUVECs.^35^ In addition, the lack of IFN-β signaling induced choroidal neovascularization in the AMD model,^40^ and deletion of *Ifnb* promoted vessel growth.^41^ In our study, we found that knockout of endothelial *Sting1* resulted in lower expression of *Ifnb1* (Figure 3H), followed by attenuated VEGFA-VEGFR2 signaling (Figure 4), which was contradictory to the anti-angiogenic effects of IFNs. Therefore, the regulation of VEGFA-VEGFR2 activity by cGAS-STING signaling is unlikely to be mediated through IFNs.

It has been reported that VEGFR2 was widely expressed by non-vascular cells, including ganglion cells and photoreceptors in the adult retina.^17^ Regarding developing retinas, VEGFR2 was mainly detected in the superficial plexus and neurons from P6 and P11 retinas.^42^^,^^43^ In addition, studies using *Vegfr2-BAC-EGFP* mice have further identified persistent up-regulation of EGFP (i.e., VEGFR2) in retinal neurons by P6, which then gradually decreased with development.^44^^,^^45^ Notably, the levels of VEGFR2 in neuroretina were even higher than in ECs, including tip cells.^45^ Consistent with the above literature, our results showed that VEGFR2 was highly expressed in neuroretina and tip cells of P7 retinas.

Targeting both cGAS-STING and VEGFA-VEGFR2 signaling may provide better efficacy, as it is reported that the STING agonist combined with anti-VEGFR2 significantly promotes vascular maturation by increasing pericyte coverage and reducing angiogenesis in tumor vasculature.^46^ Moreover, STING inhibition together with anti-VEGFA is the most potent in suppressing retinal angiogenesis in the models of choroid neovascularization and ischemic retinopathy.^26^ However, whether the combined therapy of cGAS-STING and VEGFA-VEGFR2 signaling will be optimal for treating early-stage DR awaits further future explorations.

In conclusion, this study has revealed a novel interaction of cGAS-STING signaling with VEGFA-VEGFR2 signaling in the PDR FVM, developing mouse retinas, and adult retinas across species, which indicates a vital role of cGAS-STING signaling in pathophysiological angiogenesis and vascular homeostasis.

## Materials and methods

### scRNA-seq and bulk RNA-seq databases

This study utilized public databases, including scRNA-seq data of the FVM (GSE165784^5^), adult retinas of *Homo sapiens* (GSE137846^19^ and GSE148077^20^), *Macaca* (GSE118546^22^ and GSE242229^23^), *Sus scrofa* (GSE193975^21^), and postnatal mice (P3 and P6: GSE203116^47^; P12: GSE172230^48^; P14: GSE174400^49^; P15: GSE169039^13^^,^^50^; and P26: GSE213887^51^). Analyses of bulk RNA-seq data of the PDR FVM and normal retinas (GSE60436,^52^ GSE94019^53^), *Sting1*^ΔEC^ retinas, and *Sting1*^fl/fl^ controls at P7 (Shanghai Applied Protein Technology Co., Ltd) were also conducted, and deposited at https://ngdc.cncb.ac.cn/omix/release/OMIX012206). Seurat v.4.4.0 and v.5.0.0 in R v.4.3.1 were utilized.^54^^,^^55^ Data integration and batch effect correction were performed using the Seurat CCA-based method. Specifically, integration was conducted using the top 2,000 highly variable genes. Thirty canonical components were utilized to identify integration anchors, thereby aligning shared biological structures and minimizing batch-specific effects. Subsequent steps consisting of normalization, variable gene selection, dimensionality reduction, clustering, and uniform manifold approximation and projection (UMAP) visualization were performed by using the Seurat functions.

### Cluster classification and annotation

Differentially expressed genes for cell clusters are annotated by markers from the literature for specific cell types and by the “Enrichr” software,^56^ as listed in Table S1.

### Calculation of gene signature scores

The enrichment scores were calculated by summarizing the expression levels of signature genes using the “Apply” function in R. Normalized enrichment scores and nominal *p* values were calculated by the Kolmogorov-Smirnov test. The gene signatures are listed in Table 1.

**Table 1:** Gene signature scores for signaling pathway

Signaling pathway	Gene signatures
cGAS-STING	*Irf3*, *Tmem173*, *Stat1*, *Cxcl10*, *Ifi44*, *Ifih1*, *Irf7*, *Isg15*, *Rtp4*, *Ifit1*, *Ifit2*, *Ifit3*, *Oasl2*, *Ikbke*, *Ddx58*, *Cgas*, *Tbk1*, *Icam1*, *Nfkb1*, *Stat2*
Angiogenesis	*Mmp2*, *Vegfa*, *Vegfb*, *Vegfc*, *Vegfd*, *Pigf*, *Kdr*, *Flt1*, *Nrp1*, *Nrp2*, *Ang*, *Angpt2*, *Fgf1*, *Fgf2*, *Tek*, *Notch1*, *Pdgfb*, *Tgfbr1*, *Tgfb1*, *Acvrl1*, *Id1*, *Tgfbr3*, *Efnb2*, *Efna1*, *Ephb4*, *Ephb2*, *Epha4*
Hallmark angiogenesis (from MSigDB^57^)	*Apoh*, *App*, *Ccnd2*, *Col3a1*, *Col5a2*, *Cxcl5*, *Fgfr1*, *Fstl1*, *Itgav*, *Jag1*, *Jag2*, *Kcnj8*, *Lpl*, *Lrpap1*, *Lum*, *Msx1*, *Nrp1*, *Olr1*, *Pdgfa*, *Pf4*, *Pglyrp1*, *Postn*, *Prg2*, *Ptk2*, *S100a4*, *Serpina5*, *Slco2a1*, *Spp1*, *Stc1*, *Thbd*, *Timp1*, *Tnfrsf21*, *Vav2*, *Vcan*, *Vegfa*, *Vtn*
Endothelial activation/proliferation	*Mki67*, *Cxcr4*, *Dll4*, *Aplnr*, *Esm1*

### Correlation analysis and GSEA

Spearman correlation analyses and scatterplots were performed between gene signatures of cGAS-STING and angiogenesis/VEGFA-VEGFR2 signaling, using the Ggpubr R (v.0.6.0) and Ggstatsplot R (v.0.12.1) packages. Spearman correlation analyses and correlation matrices were performed between each gene of cGAS-STING (or angiogenesis), using the corrplot R package (v.0.92). GSEA was carried out using the Gseavis R Package (v.0.0.9) to compute enrichment scores for each gene and signature.

### Cell trajectory analysis

To construct cell trajectories for retinal ECs in postnatal mice, we subgrouped these cells based on their developmental states. Pseudotime analysis was conducted and normalized using the Monocle R package (v.2.8.0) with the reverse graph embedding machine-learning algorithm.^58^ Then, the negative binomial overdispersion was estimated for each gene using the “estimateDispersions” function. The “detectGenes” counted cells expressing each feature in a CellDataSet object above a threshold of 0.1, and features detected in more than 10 cells were selected for further analysis. To identify genes that changed steadily along the identified trajectory, a likelihood ratio test for a negative binomial model was performed using the “differentialGeneTest” function. Genes that were changed significantly after multiple-hypothesis correction were used to perform dimension reduction using the DDRTree method (Monocle). A pseudotime trajectory was plotted using the “plot_cell_trajectory” on Monocle, and cells were positioned onto the trajectory using the “orderCells” function. The colors of the trajectory indicated the state, pseudotime, and cluster information.

### Generation of *Sting1*^ΔEC^ mice

The *Sting1*^fl/fl^ mice were generated by the Cyagen Biosciences Co. Ltd. (Jiangsu, China) and crossbred with endothelial-specific Tek-Cre mice to obtain *Sting1*^ΔEC^ mice, with *Sting1*^fl/fl^ littermates as controls. Genotyping was confirmed using the following primers: (1) *Sting1* forward primer: CTGAGGACACACCCTTAGGAATG, *Sting1* reverse primer: GTTTGCCTATGTGAACAAGACCATG; and (2) Tek-Cre forward primer: GGGCAGTCTGGTACTTCCAAGCT, Tek-Cre reverse primer:CTTGATTCACCAGATGCTGAGGTTA. Mice were maintained *ad libitum*, with free access to food and water under a 12-h light/dark cycle and a germ-free environment. The retinas from both male and female pups at ages P7, P14, and P21 were examined and quantified. All the procedures on mice were approved by the Institutional Animal Care and Use Committee at The Third Affiliated Hospital of Sun Yat-sen University (20200918).

### Retinal flatmounts and staining

Mice at P7, P14, and P21 were sacrificed. The eyeballs were fixed in 4% PFA for 10 min and dehydrated in 2X PBS for 20 min. Retinal flatmounts were enucleated and blocked in the Perm block medium (5% bovine serum albumin +0.3% Triton X-100) at room temperature for 1 h, followed by incubation of anti-IB4 (ThermoFisher, MA, USA) or anti-VEGFR2 (CST, MA, USA) at 4°C overnight. Slides were washed by PBST 3 times, and mounted with an anti-fade mounting medium (VectaShield, CA, USA). Images were captured under a confocal or inverted microscope, and analyses of fluorescence intensities were performed using AngioTool^59^ and Fiji software. To quantify the filopodia bursts, the images of vascular forefronts were cropped from the original images and enlarged for clearer visualization, without any processing or editing.

### Cell culture, EdU, wound closure, transwell, real-time PCR, and western blot assays

HRVECs (Otwo Biotech, Jiangsu, China) were maintained in 1 g/L of glucose DMEM medium supplemented with 10% fetal bovine serum. Overexpression of STING was achieved by incubating with a mixture containing Ad-STING (MOI: 50) and polyethyleneimine (2.5 μg/mL). *STING1*^CRISPR^ HRVECs were generated previously.^6^ BeyoCick EdU-555 (Beyotime, Shanghai, China) was incubated for 24 h before fixation for imaging. Actinomyocin-D (2 μM) was applied to block mRNA transcription. HRVECs were switched to basal culture medium without fetal bovine serum before addition of VEGFA (50 ng/mL). The wounds in the cell culture were scrapped, and images were taken after 24 h. The rate of wound closure was calculated by averaging the width of the 24-h or 8-h wound to that of the 0-h wound. The migratory capacities were also confirmed by the transwell assay. Briefly, cells were seeded inside the 8-μm Falcon transwell (Corning, NY, USA), and the transwells were plated and cultured in the 6-well plate. Cells were stained by crystal violet after 24 h; non-migrated cells were gently wiped out, and migrated cells were counted using ImageJ. Real-time PCR measurement and western blot analysis were performed according to the standard procedures, with the primer sequences and antibodies listed in Tables S2 and S3. Images were obtained by the ChemiDOC XRS + system (Bio-Rad, USA). The densitometry of the bands was semi-quantified using the Image Lab Software (Bio-Rad, USA). The ratios of p-AKT and AKT to their corresponding β-actin were calculated, and then p-AKT/β-actin was divided by AKT/β-actin and averaged for each group.

### Statistical analysis

All the results were expressed as mean ± standard error of the mean (SEM). Comparisons between two groups were performed with Student’s *t* tests. Statistical significance was set by ∗*p* < 0.05, ∗∗*p* < 0.01, and ∗∗∗*p* < 0.001.

## Data and code availability

scRNA-seq databases analyzed in this study were downloaded from the NCBI Sequence Read Archive, and the accession numbers are present in the materials and methods section or the supplemental information. The bulk RNA-seq data are available in the NGDC OMIX repository database under the accession code OMIX012206 (https://ngdc.cncb.ac.cn/omix/release/OMIX012206).

## Acknowledgments

The animal study was approved by the Institutional Animal Care and Use Committee at the Third Affiliated Hospital of Sun Yat-sen University (20200918). This work was supported by grants from the 10.13039/100017357Science and Technology Project of Guangzhou City (2024A03J0002), the 10.13039/100014718National Natural Science Foundation of China (82270886, 82300968, 82200891, and 82300901), and the 10.13039/501100012151Sanming Project of Medicine in Shenzhen (SZSM202402019).

## Author contributions

Y.C., S.L., and X.H. designed and supervised the study. R.Z. and S.L. analyzed scRNA-seq data. Z.W., H.L., H.A., and G.S. generated *Sting1*^ΔEC^ mice and *STING*^CRISPR^ cell line. X.H., R.Z., S.W., L.F., R.G., and L.Z. performed experiments, statistical analysis, and visualized results. X.H., Y.C, R.G., L.F., and L.Z. provided funding support. X.H., S.L., and Y.C. wrote and revised the manuscript. All authors have read and approved the final manuscript.

## Declaration of interests

The authors declare no competing interests.

## Supplemental information

Document S1. Figures S1–S6 and Tables S1–S3

Document S2. Article plus supplemental information
