# Supplementary material for: An atlas of cGAS-STING signaling in pathophysiological angiogenesis and retinal vascular homeostasis across species
Source: Mol Ther Nucleic Acids. 2026 Jan 24;37(1):102847. doi: 10.1016/j.omtn.2026.102847 (PMC12914536; doi:10.1016/j.omtn.2026.102847)
Supplement: Document S1. Figures S1–S6 and Tables S1–S3 [file mmc1.pdf]

OMTN, Volume *37*

Supplemental information

An atlas of cGAS-STING signaling in pathophysiological angiogenesis and retinal vascular homeostasis across species

Xuemin He, Rui Zeng, Siying Wen, Zheyao Wen, Hejun Li, Heying Ai, Rong Gao, Liwen Fan, Li Zhou, Guojun Shi, Yanming Chen, and Shasha Li

Supplemental materials

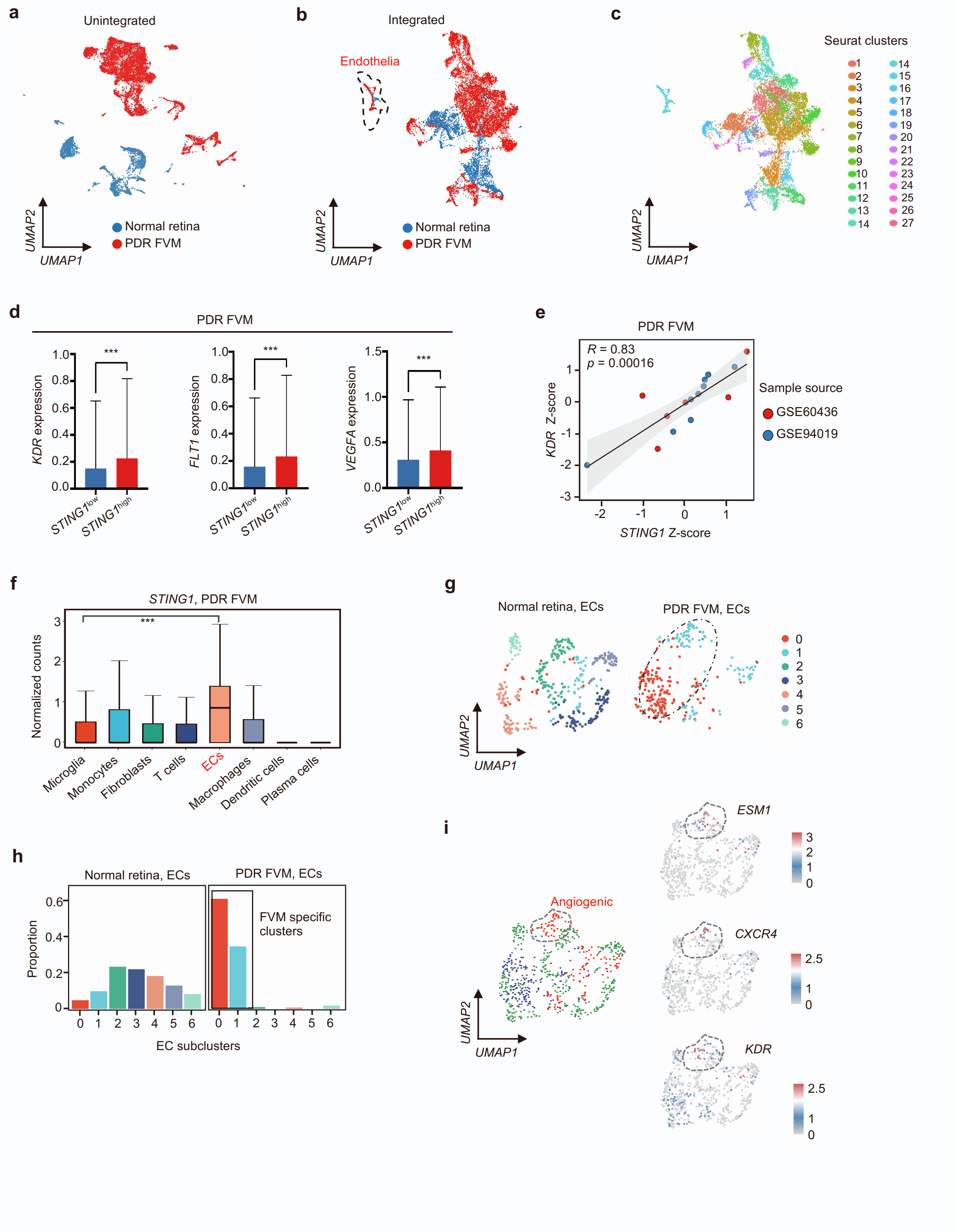

Fig. S1 | Increment of VEGF-VEGFR2 signaling in the PDR FVM. a, b UMAP plots of unintegrated and integrated cell clusters of the PDR FVM and normal retinas profiled by

scRNA-seq. **c** UMAP plots of Seurat clusters from the PDR FVM and normal retinas profiled by scRNA-seq. **d** Quantification of the mRNA levels of *KDR*, *FLT1*, and *VEGFA* in the cells from the PDR FVM and normal retinas. **e** Spearman correlation coefficient (R score) and correlation test (*p*-value) of *STING1* and *KDR* in the PDR FVM and normal retinas. **f** Box plot depicting the normalized counts of *STING1* in the cells from the PDR FVM. **g** UMAP plot of EC subclusters in normal and PDR retinas. **h** Proportion of EC subclusters in normal and PDR retinas. **i** Feature plots illustrating expression of angiogenic markers in retinal ECs. ***, $p<0.001$.

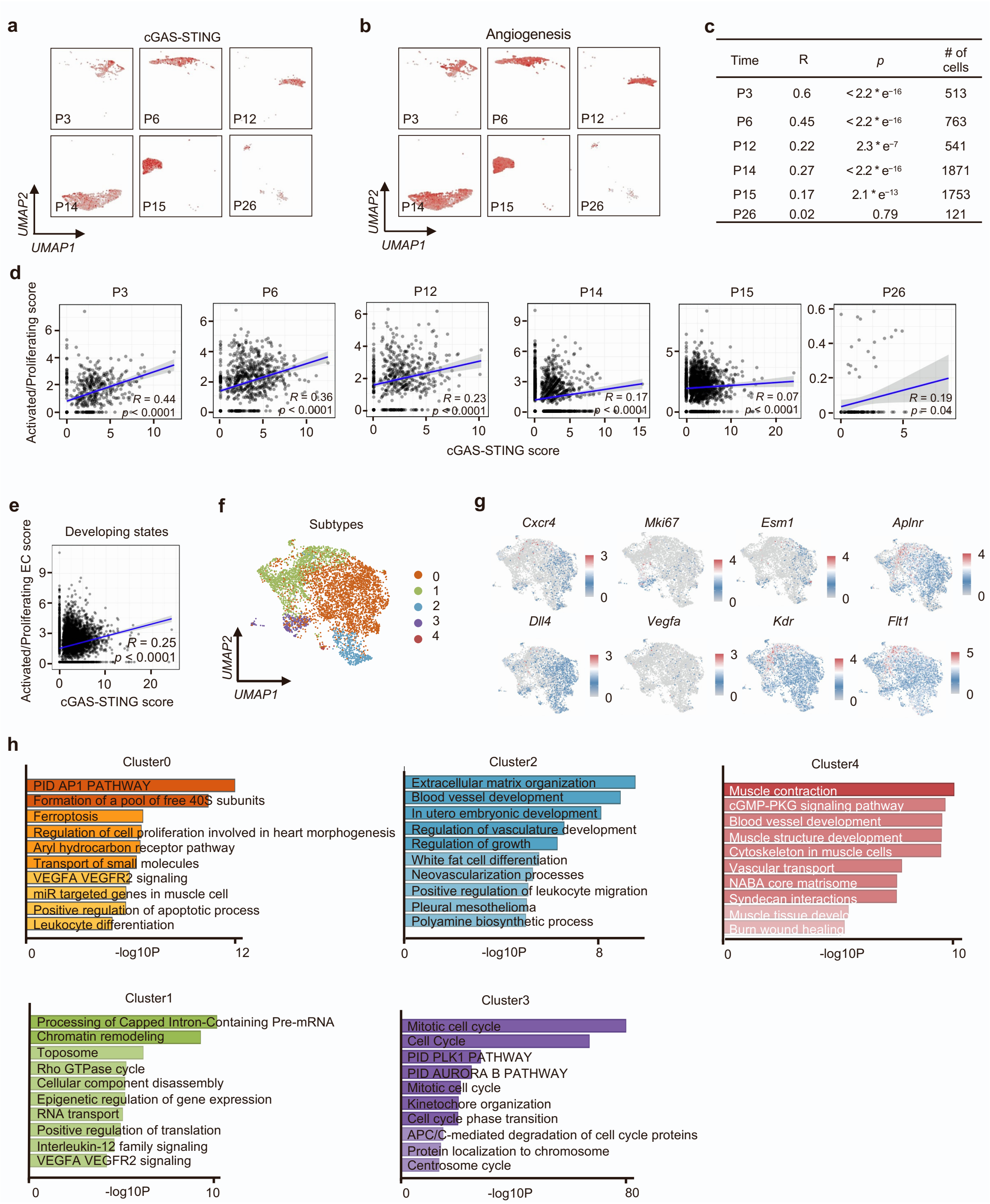

Fig. S2 | Expression levels of cGAS-STING signaling and angiogenesis in the ECs of mouse retinas across development stages. a, b UMAP plots of split projections of gene signatures for cGAS-STING signaling and angiogenesis across developmental stages. Expression levels are color-coded as: gray (not expressed) and red (expressed). **c** List of the Spearman correlation coefficient (R score) and *p*-values of angiogenesis and

cGAS-STING signaling in the retinal ECs, as well as the numbers of ECs in the mouse retinas from P3 to P26. **d, e** Correlation analyses between cGAS-STING and activated/proliferating EC signature across developmental states. **f** UMAP plot of re-clustered angiogenic ECs across developmental stages. **g** Feature plots showing representative angiogenic marker gene expression. **h** Pathway enrichment analysis of each angiogenic subcluster revealing distinct biological characteristics.

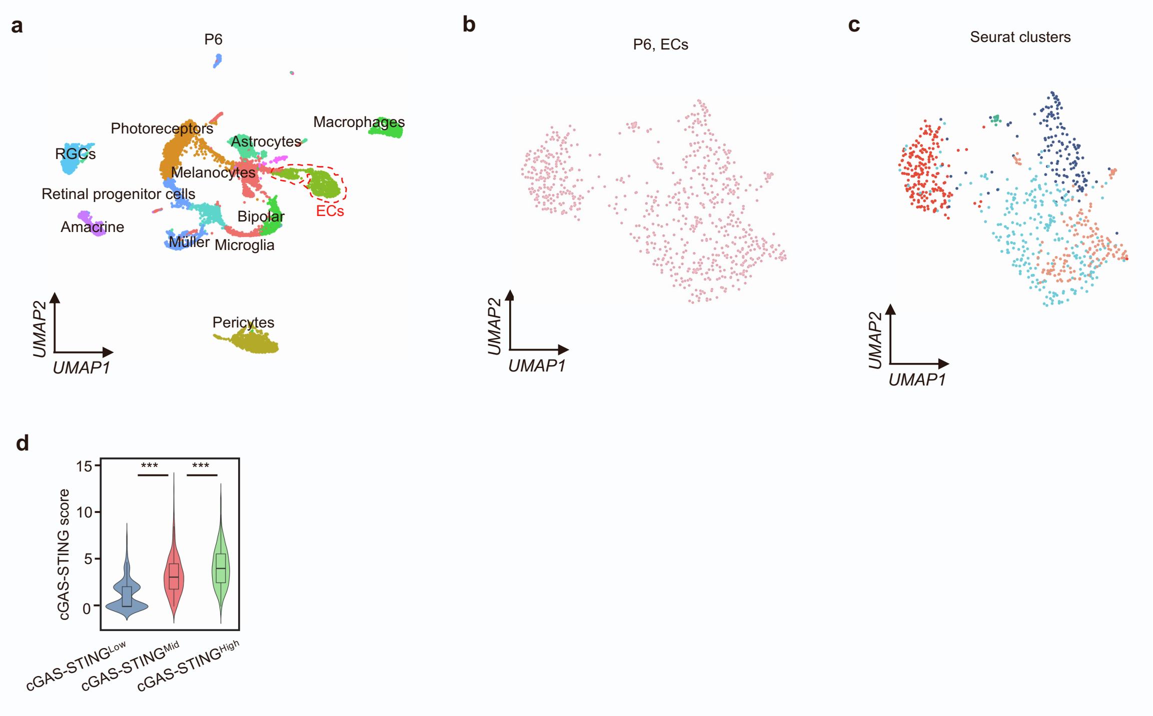

Fig. S3 | Cell clusters of the mouse retinas at P6. a, b UMAP plots of retinal cell clusters and ECs from P6 mice profiled by scRNA-seq. The red circle denotes ECs. **c** UMAP visualization of EC clusters at P6. **d** Violin plots showing cGAS-STING signaling scores in signature grouping.

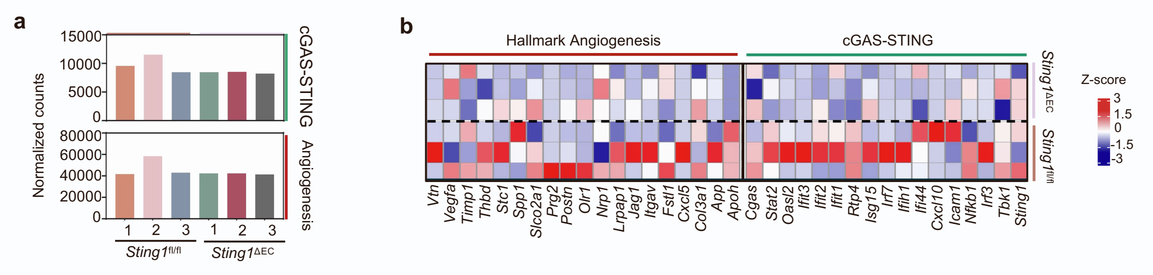

Fig. S4 | Down-regulated expression patterns of cGAS-STING signaling and hallmark angiogenesis in the retina of *Sting1*$^{\Delta EC}$ mice. a Total values of gene signature scores for cGAS-STING signaling and angiogenesis in *Sting1*$^{\Delta EC}$ and *Sting1*$^{fl/fl}$ retinas at P7. **b** Heatmap of cGAS-STING signaling and hallmark angiogenesis in *Sting1*$^{\Delta EC}$ and *Sting1*$^{fl/fl}$ retinas at P7. White arrows indicate retinal vessels, and yellow arrows indicate non-vascular areas.

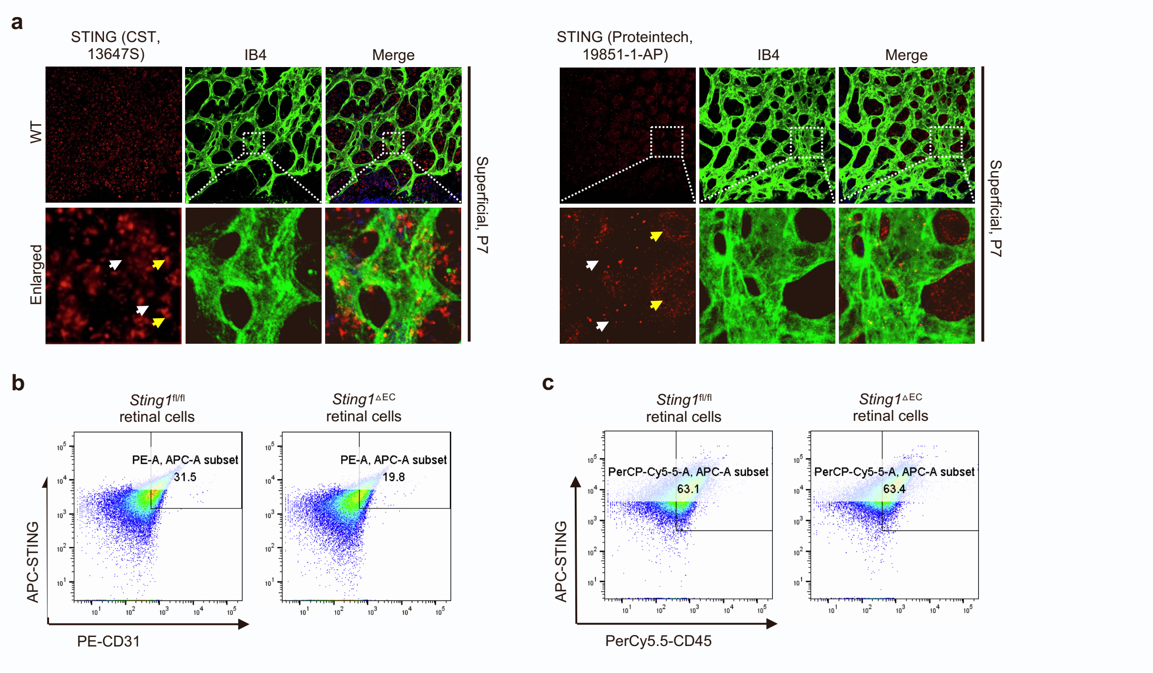

Fig. S5 | Detection of STING in mouse retinas. a Immunostaining of STING using two different antibodies in *Sting1*$^{fl/fl}$ retinas at P7. **b, c** Isolation and flow cytometry measurement of STING^{+}CD31^{+} cells and STING^{+}CD45^{+} cells from *Sting1*$^{\Delta EC}$ and *Sting1*$^{fl/fl}$

retinas.

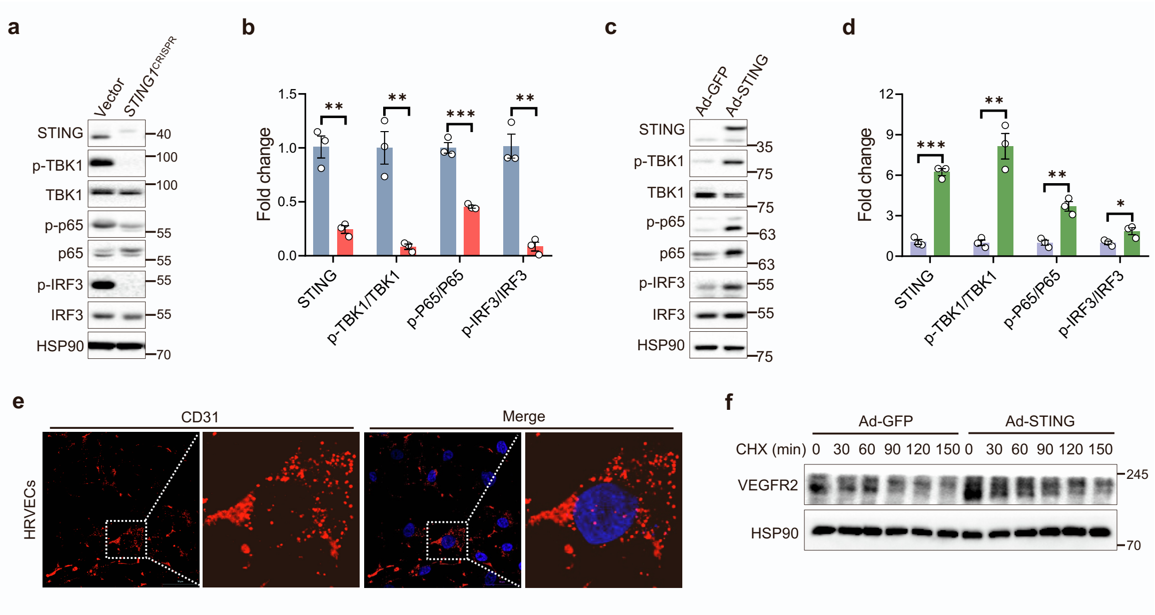

Fig. S6| Status of cGAS-STING signaling by STING modulation. a-d Western blot analysis and densitometry quantification of STING signaling components STING, p-TBK1, TBK1, p-p65, p65, p-IRF3, and IRF3 in *STING1*^{CRISPR} HRVECs and HRVECs after infection with Ad-STING for 48 h (N=3). **e** Immunostaining of CD31 in HRVECs to confirm its endothelial lineage. **f** Western blot analysis of VEGFR2 levels in HRVECs after incubation of Ad-STING for 48 h, followed by CHX (50 μg/ml) for the indicated time points. *$p < 0.05$, **$p < 0.01$, ***$p < 0.001$.

Table S1. DEGs for cell clusters in the retina

Cell clusters	DEGs
Rod photoreceptors	*Nr2e3*, *Pde6a*, *Rho*, *Gngt1*
Fetal retinal progenitors	*Onecut2*, *Vsx2*, *Lhx2*, *Ccnd1*, *Cdk4*, *Pax6*, *Ube2c*, *Ascl1*, *Fgf15*
Adult retinal progenitors	*Onecut1*, *Vsx2*, *Lhx2*, *Ccnd1*, *Cdk4*, *Pax6*, *Ube2c*, *Ascl1*, *Fgf15*
Bipolar cells	*Gng13*, *Gsg1*, *Camk2b*, *Bhlhe23*, *Grm6*, *Vsx1*, *Trnp1*, *Pcp2*
Cone photoreceptors	*Arr3*, *Chrnb4*
Müller glia	*Rlbp1*, *Slc1a3*, *Sox9*, *Glul*, *Dusp1*, *Aqp4*
Microglia	*C1qa*, *Ccl3*, *Apoe*
Retinal ganglion cells	*Nefl*, *Trh*
Amacrine cells	*Gad1*, *Tfap2a*, *Prdm13*, *Calb2*, *Slc6a9*, *Scl32a1*, *Pax6*, *Cartpt*
Endothelia	*Pecam1*, *Egfl7*, *Cldn5*, *Cdh5*, *Ackr1*
VasEndo	*Ackr1*
Pericytes	*Cspg4*, *Pdgfrb*, and *Kcnj8*
Astrocytes	*Gfap*, *Pdgfra*

Table S2. List of primer sequences for Real-time PCR measurements

Gene	Sequences
Mouse *Irf3* F	GAGAGCCGAACGAGGTTCAG
Mouse *Irf3* R	CTTCCAGGTTGACACGTCCG
Mouse *Sting1* F	TTTGCCATGTCACAGGATGC
Mouse *Sting1* R	ATGAGGCGGCAGTTATTTCG
Mouse *Ifnb1* F	GCCTTTGCCATCCAAGAGATGC
Mouse *Ifnb1* R	ACACTGTCTGCTGGTGGAGTTC

Mouse *Kdr* F	CGAGACCATTGAAGTGACTTGCC
Mouse *Kdr* R	TTCCTCACCCTGCGGATAGTCA
Mouse *Pecam1* F	CCAAAGCCAGTAGCATCATGGTC
Mouse *Pecam1* R	GGATGGTGAAGTTGGCTACAGG
Mouse *Col4a1* F	ATGGCTTGCCTGGAGAGATAGG
Mouse *Col4a1* R	TGGTTGCCCTTTGAGTCCTGGA
Mouse *Tjp1* F	GTTGGTACGGTGCCCTGAAAGA
Mouse *Tjp1* R	GCTGACAGGTAGGACAGACGAT
Mouse *Cldn5* F	TGACTGCCTTCCTGGACCACAA
Mouse *Cldn5* R	CATACACCTTGCACTGCATGTGC
Mouse *Actb* F	CATTGCTGACAGGATGCAGAAGG
Mouse *Actb* R	TGCTGGAAGGTGGACAGTGAGG
Human *STING1* F	TTCGAACTTACAATCAGCATTACAA
Human *STING1* R	CTCATAGATGCTGTTGCTGTAAACC
Human *IRF3* F	TCTGCCCTCAACCGCAAAGAAG
Human *IRF3* R	TACTGCCTCCACCATTGGTGTC
Human *TBKBP1* F	CAACCTGGAAGCGGCAGAGTTA
Human *TBKBP1* R	ACCTGGAGATAATCTGCTGTCGA
Human *NFKB1* F	ATGTGGAGATCATTGAGCAGC
Human *NFKB1* R	CCTGGTCCTGTGTAGCCATT
Human *KDR* F	GGCCCAATAATCAGAGTGGCA
Human *KDR* R	CCAGTGTCATTTCCGATCACTTT
Human *FLT1* F	TTTGCCTGAAATGGTGAGTAAGG
Human *FLT1* R	TGGTTTGCTTGAGCTGTGTTC
Human *VEGFA* F	AGGGCAGAATCATCACGAAGT
Human *VEGFA* R	AGGGTCTCGATTGGATGGCA
Human *ACTB* F	CACCATTGGCAATGAGCGGTTC

Human *ACTB* R	AGGTCTTTGCGGATGTCCACGT

Table S3. List of antibodies

Antibodies	Company	Catalog number
anti-CD31	Cell Signaling Technology	3528S
anti-VE-cadherin	Cell Signaling Technology	2500S
anti-Phospho-TBK1 (Ser172)	Cell Signaling Technology	5483S
anti-TBK1	Cell Signaling Technology	38066S
anti-Phospho-p65 (Phospho Ser536)	Cell Signaling Technology	3033S
anti-p65	Cell Signaling Technology	8242S
anti-Phospho-IRF3 (Phospho Ser386)	Abcam	ab76493
anti-IRF3	Cell Signaling Technology	4302S
anti-STING	Cell Signaling Technology	13647S
anti-STING	Proteintech	19851-1-AP
Anti-CD31	Abclonal	A19014
anti-HSP90	Abcam	ab13492
anti-p-VEGFR2	Cell Signaling Technology	3770S
anti-VEGFR2	Cell Signaling Technology	9698S
anti-VEGFA	Cell Signaling Technology	65373
anti-Phospho-AKT (Phospho Ser473)	Cell Signaling Technology	4060S
anti-AKT	Cell Signaling Technology	4691S

anti-Phospho-ERK1/2 (Phospho Thr202/Tyr204)	Cell Signaling Technology	4370S
anti-ERK1/2	Cell Signaling Technology	4695S
anti-Isolectin GS-IB4	ThermoFisher	I21411
Goat anti-mouse HRP	BIO-Rad	1706516
Goat anti-rabbit HRP	BIO-Rad	1706515
anti-rabbit (Alexa Fluor 647)	Cell Signaling Technology	4414S
anti-rabbit (Alexa Fluor 647)	Cell Signaling Technology	4409S
